# The effect of 100% single-occupancy rooms on acquisition of extended-spectrum beta-lactamase-producing Enterobacterales and intra-hospital patient transfers: a prospective before-and-after study

**DOI:** 10.1186/s13756-022-01118-7

**Published:** 2022-06-02

**Authors:** Adriënne S. van der Schoor, Juliëtte A. Severin, Anna S. van der Weg, Nikolaos Strepis, Corné H. W. Klaassen, Johannes P. C. van den Akker, Marco J. Bruno, Johanna M. Hendriks, Margreet C. Vos, Anne F. Voor in ’t holt

**Affiliations:** 1 Department of Medical Microbiology and Infectious Diseases, Erasmus MC University Medical Center, Rotterdam, The Netherlands; 2 Department of Intensive Care Adults, Erasmus MC University Medical Center, Rotterdam, The Netherlands; 3 Department of Gastroenterology and Hepatology, Erasmus MC University Medical Center, Rotterdam, The Netherlands; 4 Department of Surgery, Erasmus MC University Medical Center, Rotterdam, The Netherlands

**Keywords:** Private room, Extended-spectrum beta-lactamase, Enterobacteriaceae, Enterobacterales, Acquisition, Patient transfers

## Abstract

**Background:**

Extended-spectrum beta-lactamase-producing Enterobacterales (ESBL-E) are a well-known cause of healthcare-associated infections. The implementation of single-occupancy rooms is believed to decrease the spread of ESBL-E. Additionally, implementation of single-occupancy rooms is expected to reduce the need for intra-hospital patient transfers. We studied the impact of a new hospital with 100% single-occupancy rooms on the acquisition of ESBL-E and on intra-hospital patient transfers.

**Methods:**

In 2018, the Erasmus MC University Medical Center moved from an old, 1200-bed hospital with mainly multiple-occupancy rooms, to a newly constructed 522-bed hospital with 100% single-occupancy rooms. Adult patients admitted between January 2018 and September 2019 with an expected hospitalization of ≥ 48 h were asked to participate in this study. Perianal samples were taken at admission and discharge. Patient characteristics and clinical information, including number of intra-hospital patient transfers, were collected from the patients’ electronic health records.

**Results:**

Five hundred and ninety-seven patients were included, 225 in the old and 372 in the new hospital building. Fifty-one (8.5%) ESBL-E carriers were identified. Thirty-four (66.7%) patients were already positive at admission, of which 23 without recent hospitalization. Twenty patients acquired an ESBL-E, seven (3.1%) in the old and 13 (3.5%) in the new hospital building (*P* = 0.801). Forty-one (80.4%) carriers were only detected by the active screening performed during this study. Only 10 (19.6%) patients, six before and four during hospitalization, showed ESBL-E in a clinical sample taken on medical indication. Fifty-six (24.9%) patients were transferred to other rooms in the old hospital, compared to 53 (14.2%) in the new hospital building (*P* = 0.001). Intra-hospital patient transfers were associated with ESBL-E acquisition (OR 3.18, 95%CI 1.27–7.98), with increasing odds when transferred twice or more.

**Conclusion:**

Transitioning to 100% single-occupancy rooms did not decrease ESBL-E acquisition, but did significantly decrease the number of intra-hospital patient transfers. The latter was associated with lower odds on ESBL-E acquisition. ESBL-E carriers remained largely unidentified through clinical samples.

**Trial registration:**

This study was retrospectively registered in the Dutch National Trial Register on 24-02-2020, with registration number NL8406.

**Supplementary Information:**

The online version contains supplementary material available at 10.1186/s13756-022-01118-7.

## Introduction

Highly resistant microorganisms (HRMO) are a common cause of healthcare-associated infections (HAI), and are a worldwide threat to public health and modern healthcare [1]. Among HRMO, extended-spectrum beta-lactamase-producing Enterobacterales (ESBL-E) are most frequently identified. Worldwide, the prevalence of ESBL-E in the community differs from 2 to 46% [2]. In hospitals, this prevalence is higher and outbreaks with ESBL-E occur. Hospital design is thought to play an essential role in the spread of HRMO including ESBL-E [3–5]. To decrease the spread of HRMO within hospitals, the Facility Guideline Institute recommends transitioning to 100% single-occupancy rooms for medical/surgical units [6]. Moreover, their 2018 report advises 100% single patient rooms in adult critical care units [7]. An added benefit of single-occupancy rooms is that they remove the necessity for intra-hospital patient transfers for small procedures, social circumstances (*e.g.* end-of-life care), or for an indication of contact isolation [8]. By reducing the number of intra-hospital patient transfers, which leads to less exposure of the patient to different hospital environments, and by reducing the exposure to unidentified infected or colonized roommates, the implementation of 100% single-occupancy rooms is expected to reduce the risk of HRMO acquisition and transmission [9]. However, current literature shows conflicting results for the effect of single-occupancy rooms on the acquisition of HRMO [4, 10, 11]. Furthermore, literature on the effect of single-occupancy rooms on ESBL-E acquisition is limited to the comparison of ESBL-E acquisition between an intensive care unit (ICU) with an open plan and an ICU with single-occupancy rooms, which showed no significant difference [11].

In May 2018, the Erasmus MC University Medical Center (Erasmus MC) relocated from an old hospital building, with mainly multiple-occupancy rooms, to a newly constructed hospital building with 100% single-occupancy rooms. We used this unique opportunity to determine the effect of relocating to a new hospital with 100% single-occupancy rooms on the acquisition of ESBL-E by determining ESBL-E carriage in patients at admission and discharge in both buildings. Whole genome sequencing (WGS) was used to determine if strains at discharge were identical to those present at admission or the result of acquisition during hospitalization. Additionally, we aimed to determine the effect of intra-hospital patient transfers on ESBL-E acquisition, and to identify the percentage of ESBL-E carriers that remained undetected by clinical samples.

## Methods

### Study design and setting

This study was performed at the Erasmus MC, a university medical center located in Rotterdam, the Netherlands. On May 18, 2018, the adult clinic of the Erasmus MC relocated from an old, 1200-bed hospital building with mainly multiple-occupancy rooms and shared bathrooms, to a newly constructed 522-bed hospital building with 100% single-occupancy rooms and private bathrooms. To determine the prevalence of colonization with ESBL-E and the incidence of acquisition of ESBL-E in the old and new hospital building, a prospective before-and-after study was performed. Participating departments were cardiology, gastroenterology and hepatology, general surgery, hematology, adult ICU, internal medicine, nephrology, neurology, neurosurgery, orthopedics, and plastic surgery, which do not always correspond to the admission specialization of the patients.

### Room types

In the old building, almost all departments consisted of two- and four-patient rooms, and bathrooms were shared, with an average of four patients per toilet (range four to seven) and seven patients per shower (range five to nine). Exceptions were the isolation department, the adult ICU, and three hematology departments. The isolation department consisted of solely single-occupancy rooms with anterooms and private bathrooms, and the ICU consisted of solely single-occupancy rooms, some with anterooms but all without bathrooms. The three hematological departments consisted of 83.3, 80.0 and 69.2% single-occupancy rooms and private bathrooms. All multiple-occupancy rooms, two- or three-patient rooms, had attached shared bathrooms. Two of the hematology wards were located at another location in Rotterdam; the Erasmus MC Cancer Institute, location Daniel den Hoed. The Cancer Institute also relocated to the new hospital building on the same day. In the new hospital building, all departments consisted of only single-occupancy rooms with private bathrooms, with anterooms for hematology and isolation rooms.

### Patient inclusion

From January 1, 2018 until September 1, 2019, all adult patients with an expected hospital stay of ≥ 48 h admitted to participating departments were asked to participate. Additionally, patients needed to understand and read Dutch. Patients who were admitted in the weekend or on holidays, via the emergency room, or who were cared for in airborne isolation were not approached for participation, as well as patients who were legally incapable in making decisions regarding participating, or patients who were in end-of-life stage. Patients with multiple hospitalizations during the study period were allowed to participate more than once. No additional information on HRMO risk factors were obtained before including patients (*i.e.* non-targeted screening). After obtaining written informed consent, perianal samples were collected within 24 h of admission, and on the day of discharge from the hospital. Patients who were admitted to the ICU during their hospital stay were considered as new admissions, even when they were already included in the study. Admission samples were taken on the day of admission to the ICU and discharge samples on the day patients were discharged from the ICU. Samples were either taken by trained members of the research team or patients could self-sample with clear verbal instructions of the members of the research team. Patients missed at discharge (*e.g.* unforeseen earlier discharge) received a letter asking them to take the sample at home, as well as a swab, swab-instructions with clear pictures and directions, and return-envelope. Patients admitted during the relocation of the hospital were asked for an additional swab, one day before relocation of the hospital. That sample was both the discharge sample for the old hospital building, and the admission sample for the new hospital building. ESBL-E colonization was defined as having a positive sample at admission. ESBL-E acquisition was defined as having a negative sample at admission and a positive sample for ESBL-E at discharge. It was also considered acquisition when patients were positive for a different ESBL-E at discharge. A different ESBL-E was defined as either being positive for a different microorganism, or when WGS showed that the discharge isolate was not identical to the admission isolate. Results of the perianal sample were not communicated to medical staff or patients, were not registered in the electronic health records (EHR), and hence, no infection prevention measures were taken based on the results, as stated in the protocol approved by the medical ethical research committee of the Erasmus MC (MEC-2017-1011).

### Microbiological methods

Perianal samples were taken with flocked swabs and transported in the accompanying 1 mL Amies medium (e-Swabs (Copan Italia, Brescia, Italy)). Perianal samples collected from January 1, 2018, until January 19, 2019, were stored in a − 80 °C freezer before being processed. To prevent freezing/defrosting damage, 0.2 mL 99% glycerol was added to the samples before freezing [12]. Samples taken after January 19, 2019 were processed directly. All samples, regardless of being frozen, were processed following the same protocol. Samples were vortexed for 10 s before 250µL of the sample was inoculated in a tryptic soy broth with vancomycin (50 mg/L) and incubated overnight at 35 °C. A *Brillance*™ ESBL Agar (Oxoid, Basingstoke, UK) was inoculated from the broth with a 10 µl loop and incubated twice overnight at 35 °C. Colonies were identified to species level using Matrix-Assisted Laser Desorption/Ionization Time-Of-Flight mass spectrometry (MALDI-TOF [Bruker Daltonics, Bremen, Germany]) and antibiotic susceptibility was tested with the VITEK®2 (bioMérieux, Marcy l’Etoile, France). Antibiotic susceptibility results were interpreted according to the European Committee on Antimicrobial Susceptibility Testing (EUCAST) guidelines [13]. All ESBL-E isolates were stored in a − 80 °C freezer.

### Whole genome sequencing

WGS was performed for all identified ESBL-E isolates. Total genomic DNA was extracted using the MagNA Pure 96 platform (Roche Applied Science, Mannheim, Germany). Genomic DNA was fragmented by shearing to a size of ~ 350 bp. Libraries were prepared using the NEBNext® DNA Library Prep kit (New England Biolabs, Ipswich, MA, USA)and subjected to 150 bp paired-end sequencing creating > 100 × coverage using Illumina technology (Novogene, HongKong, China). De novo genomic assemblies were generated using Unicycler v0.4 with default parameters [14, 15]. Antimicrobial resistance (AMR) genes were detected and identified using a stand-alone version of RGI v5.1.0 based on the CARD database v3.0.5 (including perfect and strict hits) [16]. The core genome multi locus sequence type (cgMLST) was determined based on each species’ corresponding MLST scheme (https://cgmlst.org/ncs) using SeqSphere + software (Ridom, Munster, Germany). Heatmaps were performed without clustering and grouped by patients in R (https://www.R-project.org).

### Data collection

Patient characteristics were collected from the EHR, including the demographic variables age at admission and sex. For the hospitalization period, data on admission specialization, all antibiotic usage, surgical procedures, ICU admission, length of hospital stay, and number of intra-hospital patient transfers were collected. Intra-hospital transfers were defined as being transferred to another patient room for ≥ 4 h, and did not include transfers to *e.g.* the ICU, radiology, the operating theater, or the Post Anesthesia Care Unit, since the necessity of these transfers was not impacted by the transition to 100% single-occupancy rooms. Data on history of ESBL-E carriage up to 2013, bacteriological data of ESBL-E identified from clinical samples during hospitalization, and results of the hospital HRMO-screening risk-assessment score on admission was collected. This risk-assessment was performed and registered within the first 24-h of hospitalization for every patient admitted to the hospital [17, 18]. When patients were at risk according to the risk assessment, (*e.g.* having been admitted at a hospital abroad in the last 2 months; the complete assessment can be found in Additional file 3) cultures were taken and the patient was pre-emptively cared for in isolation until the results of the HRMO cultures were known [17, 18]. Finally, to illustrate the exposure to the hospital environment, we calculated the square meters (m^2^) of patient rooms and bathrooms to which patients were exposed to in the old and new hospital setting (Additional file 1).

### Statistical analyses

Patients were divided into three categories based on their admission specialization; medical, surgical or hematological. Medical patients were admitted to the specializations dermatology, endocrinology, geriatrics, immunology, infectious diseases, general internal medicine, gastroenterology and hepatology, nephrology, neurology, internal oncology, pain relief, radiology, or vascular medicine. Surgical patients were admitted to the specializations general, gastrointestinal, neurological, oncological, orthopedic, plastic, trauma, transplantation, or vascular surgery. Descriptive analyses were performed separately for these groups. For continuous variables, medians with range were presented. Normal distributed variables were analyzed with independent sample *t*-tests. The calculated m^2^ patients were exposed to were logarithmically-transformed and analyzed with independent sample *t*-tests. Categorical variables were presented as percentages and analyzed using a Chi-squared test. All *P-*values < 0.05 were considered statistically significant. To determine correlations between variables, logistic regression analyses were performed and presented with odd ratios (OR) and 95% confidence intervals (95%CI). Continuous determinants in logistic regression analyses were categorized into four categories based on quartiles. When the 95%CI did not include 1.00, it was considered statistically significant. IBM Statistical Package for the Social Sciences Solutions (SPSS) version 25 (IBM Corp., Armonk, New York, USA) was used for all analyses.

## Results

### Inclusion study samples

**Fig. 1:**
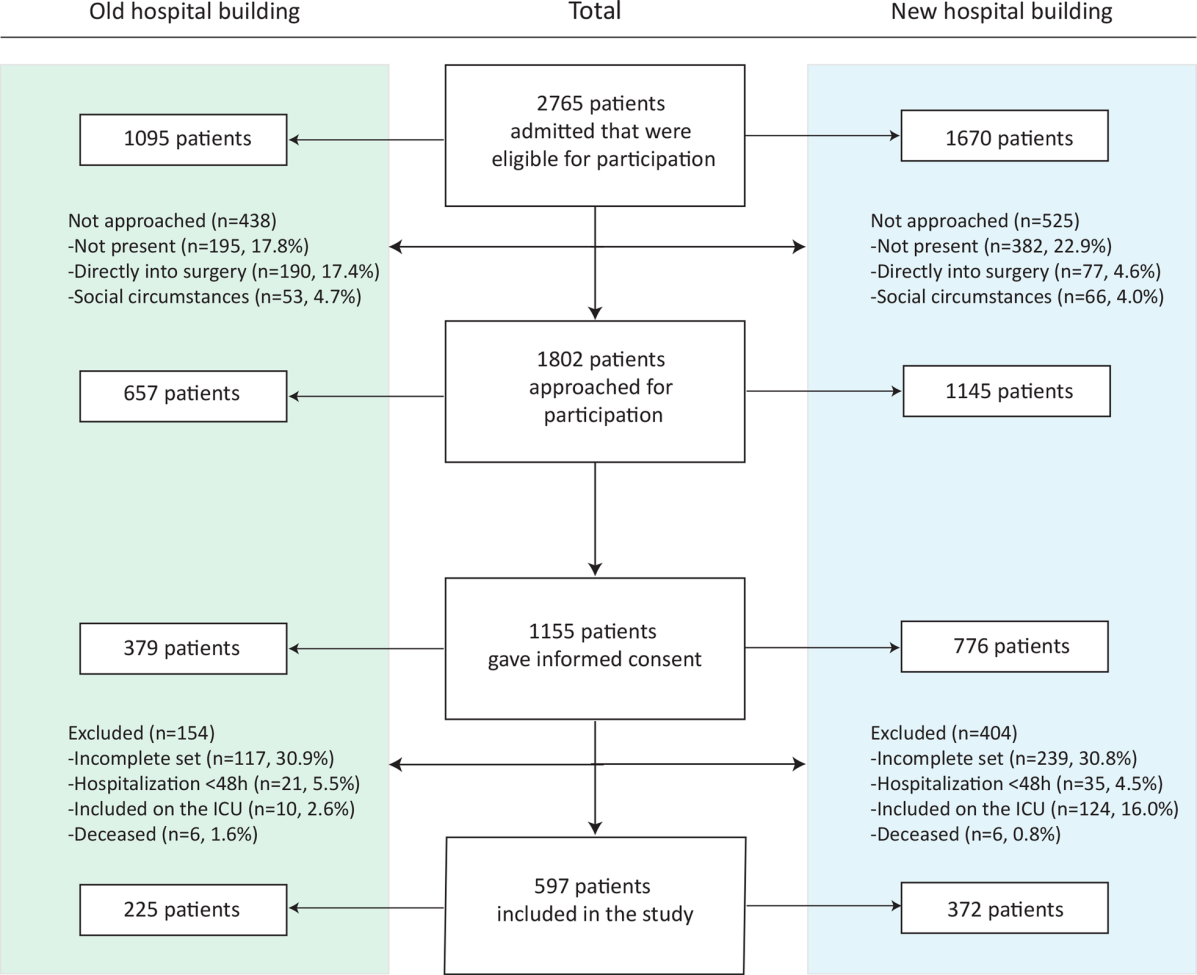
Flowchart of the inclusion of patients

In total, 1095 patients in the old building, and 1670 patients in the new building were eligible for participation in the study (Fig. 1). Patients were not approached when they were in end of life stage, or when they were legally incapable to make a decision about participating (Fig. 1). In total, 1155 patients participated in the study, 379 (32.8%) in the old and 776 (67.2%) in the new building. Due to the unexpected result that samples of patients included on the ICU were incomplete (*i.e.* missing an admission or discharge sample*)* for all patients in the old building (n = 10) and nearly all patients in the new building (107 out of 124, 86.3%), all patients included on the ICU were excluded for further analysis (Fig. 1). After exclusions, 225 out of 379 (59.4%) patients in the old building, and 372 out of 776 (47.9%) patients in the new building were included (Fig. 1). In total, 511 patients were missed at discharge and received a self-sample request at their home address. Two-hundred and sixty (50.9%) patients returned a sample, with a median return time of eight days (2–45), 251 (49.1%) patients did not return a sample and were consequently excluded. Fifteen patients were included multiple times. In the old building, four patients were admitted twice, and in the new building eight patients were included twice and three patients were included three times. Four patients were admitted during the relocation of the hospital and were thus included in both the old and the new building. The majority of patients were admitted to a surgical department, 161 (71.6%) patients in the old building and 187 (50.2%) in the new building (Table 1). The proportion of patients admitted to a medical, surgical, and hematology specialization differed between the old building and the new hospital building (15.1 vs 21.2%, 71.5 vs 50.3%, and 13.3 vs 28.5%, respectively). Univariate analyses showed no statistically significant differences in patient characteristics of patients admitted to the old building and the new building (Table 1).

**Table 1:** Characteristics of included medical, surgical, and hematological patients

Characteristics	Medical			Surgical			Hematological		
	Old building (n = 34)	New building (n = 79)	*P-*value	Old building (n = 161)	New building (n = 187)	*P-*value	Old building (n = 30)	New building (n = 106)	*P-*value
Male gender (%)	21 (61.8)	40 (50.6)	0.276	82 (50.9)	102 (54.5)	0.501	19 (63.3)	55 (51.9)	0.266
Age, median (range)	60 (19–78)	58 (20–89)	0.723	60 (18–85)	65 (24–87)	0.064	59 (33–76)	62 (20–81)	0.373
Dutch origin (%)*	28 (82.4)	68 (86.1)	0.612	143 (88.8)	164 (87.7)	0.431	26 (86.7)	97 (91.5)	0.332
Length of hospital stay, median (range)	3 (2–41)	4 (2–21)	0.374	5 (2 -43)	5 (2–72)	0.243	9.5 (2–52)	10.5 (2–146)	0.916
Surgery during hospitalization (%)	6 (17.6)	17 (21.5)	0.639	159 (98.8)	179 (95.7)	0.091	1 (3.3)	7 (6.6)	NA
ICU admission during hospitalization (%)	1 (2.9)	2 (2.5)	NA	20 (12.4)	14 (7.5)	0.122	0 (-)	2 (1.9)	NA
Antibiotic use during hospitalization (%)	14 (41.2)	27 (34.2)	0.478	147 (91.3)	171 (91.4)	0.963	26 (86.7)	92 (86.8)	0.986

*ICU* intensive care unit. *NA* not applicable, *P-*values could not be calculated due to observed and expected values below 5 for one or both groups

^*^9 patients had missing data on country of origin

### Carriage and acquisition of ESBL-producing Enterobacterales

Fifty-one out of 597 (8.5%) patients had at least one study sample positive for an ESBL-E, 16 out of 225 (7.1%) patients in the old building and 35 out of 372 (9.4%) patients in the new building (*P* = 0.330). Thirty-four patients were ESBL-E colonized at admission, 10 (4.4%) patients in the old building, compared to 24 (6.5%) in the new building (*P* = 0.305) (Table 2). Eleven out of 34 (32.4%) patients had been hospitalized in our hospital during the previous year, 23 (67.6%) patients were not hospitalized. Twelve patients, five (9.8%) in the old hospital building and 7 (13.7%) in the new hospital building, were positive at admission, but negative at discharge (*P* = 0.774). In total, 20 (3.4%) patients, seven (3.1%) in the old building and 13 (3.5%) in the new building, acquired an ESBL-E during hospitalization (*P* = 0.801) (Table 2). In total, 17 (3.0%) patients, six (2.7%) patients in the old building and 11 (3.0%) in the new building, were positive only at discharge. Additionally, one patient in the old building and one patient in the new building were positive for a different ESBL-E at discharge and one patient in the new building acquired an additional ESBL-E. *E. coli* and *K. pneumoniae* were most prevalent, at admission and discharge, and were also the ESBL-E most often acquired.

**Table 2:** Number of patients who were positive for ESBL-producing Enterobacterales at admission, at discharge, and the number of patients who acquired an ESBL-producing Enterobacterales

	Old hospital building (n = 225)			New hospital building (n = 372)		
	Admission (%)	Discharge (%)	Acquisition (%)^4^	Admission (%)	Discharge (%)	Acquisition (%)
No ESBL-E	215 (95.6)	214 (95.1)	NA	348 (93.5)	344 (92.5)	NA
ESBL-E^1,2^	10 (4.4)	11 (4.9)	7 (3.1)	24 (6.4)	28 (7.5)	13 (3.2)
*Escherichia coli*^*3*^	6 (2.7)	8 (3.5)	5 (2.2)	19 (5.1)	22 (5.9)	8 (2.2)
*Klebsiella pneumoniae*	1 (0.4)	3 (1.3)	2 (0.9)	2 (0.5)	5 (1.3)	3 (0.8)
*Citrobacter freundii*	2 (0.9)	0 (–)	NA	0 (−)	1 (0.3)	1 (0.3)
*Proteus vulgaris*	0 (–)	0 (–)	NA	2 (0.5)	0 (–)	NA
*Enterobacter cloacae* complex	0 (–)	0 (–)	NA	1 (0.3)	0 (–)	NA
*Proteus hauseri*	1 (0.4)	0 (–)	NA	0 (–)	0 (–)	NA
*Morganella morganii*	0 (–)	1 (0.4)	1 (0.4)	0 (–)	0 (–)	NA
*Klebsiella aerogenes*	0 (–)	0 (–)	NA	0 (–)	1 (0.3)	1 (0.3)

*ESBL* extended-spectrum beta-lactamase, *ESBL-E* extended-spectrum beta-lactamase producing Enterobacterales, *NA* not applicable

^1^Five patients in the old building, and seven patients in the new building were ESBL-E positive at admission and ESBL-E negative at discharge

^2^Non-significant difference between the old hospital setting and the new hospital setting for admission (*P* = 0.305), for discharge (*P* = 0.206), and for acquisition (*P* = 0.801)

^3^Non-significant difference between the old hospital setting and the new hospital setting for admission (*P* = 0.149), for discharge (*P* = 0.156), and for acquisition (*P* = 0.901)

^4^One patient was positive at admission but acquired a different ESBL-E during hospitalization and one patient acquired two ESBL-E in the old building. Consequently, there are seven patients who acquired an ESBL-E during hospitalization in the old building, but eight different ESBL-E

### Intra-hospital patient transfers and exposure to square meters

One hundred and eight out of 597 (18.1%) patients were transferred during hospitalization. Fifty-six (24.9%) patients in the old building were transferred, compared to 52 (14.0%) in the new building (*P* = 0.001). The number of patients not being transferred during hospitalization increased significantly for both medical (*P* = 0.003) and hematological patients (*P* < 0.001) in the new building (Table 3). Seventy-six out of 597 (12.7%) patients were transferred once, 42 (18.7%) in the old building and 34 (9.0%) in the new building (*P* = 0.001). The number of medical patients transferred once decreased significantly (*P* = 0.018) in the new building (Table 3). A decrease was also seen in the number of surgical and hematological patients transferred once, although not significantly (Table 3). Thirty-two (5.4%) patients were transferred at least twice, 14 (6.2%) patients in the old building and 18 (4.7%) in the new building (*P* = 0.467). This decrease was seen for both medical and hematological patients, but not for surgical patients (Table 3).

**Table 3:** Number of intra-hospital patient transfers for medical, surgical and hematological patients

	Medical			Surgical			Hematological		
	Old building (n = 34)	New building (n = 79)	*P-*value	Old building (n = 161)	New building (n = 187)	*P-*value	Old building (n = 30)	New building (n = 106)	*P-*value
Not transferred	22 (64.7)	70 (88.6)		127 (78.9)	152 (81.3)		20 (66.7)	98 (92.5)	
Transferred	12 (35.3)	9 (11.4)	**0.003**	34 (21.1)	35 (18.7)	0.575	10 (33.3)	8 (7.5)	< **0.001**
1	8 (23.5)	6 (7.6)	**0.018**	29 (18.0)	21 (11.2)	0.072	5 (16.7)	7 (6.6)	0.086
≥ 2	4 (11.8)	3 (3.8)	NA	5 (3.1)	14 (7.5)	0.073	5 (16.7)	1 (0.9)	NA

*OR* odds ratio, 95% *CI* 95% confidence interval, *ESBL-E* extended-spectrum beta-lactamase producing Enterobacterales *NA* not applicable, *P-*values could not be calculated due to observed and expected values below 5 for one or both groups. *P*-values in bold are significant

^a^Information about the overall number of patients positive for ESBL-E at admission and discharge, and patients who acquired an ESBL-E during hospitalization can be found in Table 2

In the new building, patients were exposed to less m^2^ during hospitalization than in the old building. Overall, the median m^2^ patients were exposed to in the old building was 43.3 m^2^ (21.9–177.9), compared to 22.9 m^2^ (22.9–114.6) in the new building (*P* < 0.001) (Additional file 1).

### Intra-hospital patient transfers and acquisition of ESBL-E

Eight out of 108 (7.4%) transferred patients acquired an ESBL-E, compared to 12 out of 489 (2.3%) patients that were not transferred (OR 3.18, 95%CI 1.27–7.98). Five out of 32 (15.6%) patients that were transferred twice or more acquired an ESBL-E, compared to 15 out of 565 (2.7%) patients who were once or not transferred (OR 6.79, 95%CI 2.29–20.06). Patients who were transferred once did not have significantly higher odds for ESBL-E acquisition (OR 1.22, 95%CI 0.35–4.26). Having a hospitalization period of six to ten days was associated with higher odds on having intra-hospital patient transfers, compared to patients admitted two or three days (OR 3.01, 95%CI 1.53–5.91), as well as patients hospitalized ten days or longer (OR 3.75, 95%CI 1.97–7.14). Patients whom acquired an ESBL-E during hospitalization had a median length of stay of nine days (2–146), patients who did not acquire an ESBL-E had a median length of stay of 6 days (2–72). No significant association was identified between length of hospitalization and acquisition of ESBL-E.

### Core genome MLST and detection of AMR genes of ESBL-producing *E. coli* and *K. pneumoniae*

**Fig. 2:**
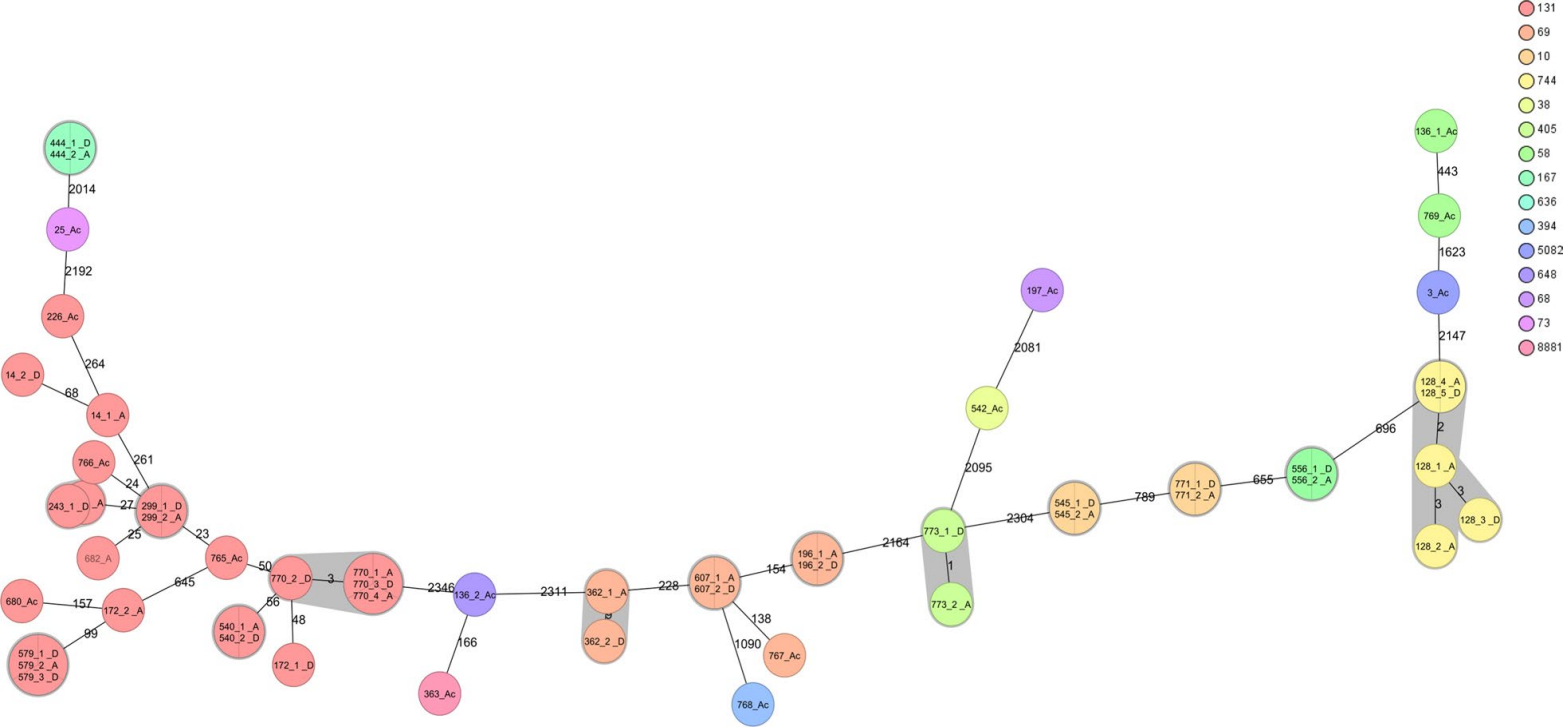
Core genome MLST analysis based on 2513 loci of *E. coli* isolates from patients positive at admission and discharge, and of *E. coli* isolates acquired during hospitalization. Node numbers represent isolate numbers and line numbers show the number of different alleles between the isolates. A cut-off value of > 10 alleles difference (embedded in the SeqSphere software) was applied to consider strains to be different. Colors match the sequence types (ST). A: Admission, D: Discharge and Ac: Acquired

WGS was performed on all 82 strains isolated from 51 patients. The majority of strains were ESBL-producing *E. coli* isolates (61 strains from 39 patients) and ESBL-producing *K. pneumoniae* (12 strains from 7 patients). The *E. coli* isolates could be classified to 20 different sequence types (ST), with ST131 being the most frequently found (28, 39.4%) (Additional file 2). Patients positive for ESBL-producing *E. coli* at admission and discharge had identical STs, indicating persistent carriage. However, the discharge strains of 2 patients (172 and 14) were not identical to the admission strain, indicating acquisition during hospitalization (Fig. 2). Of the patients who acquired an ESBL-producing *E. coli*, one patient (136) acquired two different strains (Fig. 2). For *K. pneumoniae*, patients who were positive at both admission and discharge had identical strains at both moments. Detection of AMR genes confirmed the presence of beta-lactamases for all *E. coli* and *K. pneumoniae* strains, as well as the presence of other AMR genes. Detailed information on AMR genes can be found in Additional file 2.

### HRMO risk assessment and unidentified carriers

The HRMO risk-assessment questions on admission were asked to 200 (88.9%) patients in the old hospital setting, compared to 341 (91.7%) in the new hospital setting (*P* = 0.259). Six patients had a positive risk assessment, which led to pre-emptive isolation and active surveillance cultures taken in 100%. Five patients were known HRMO carriers, one patient had been admitted to a hospital abroad, but no HRMO were identified in cultures from this patient. Of the 51 ESBL-E carriers identified in our study, 49 (96.1%) carriers were not identified through the HRMO risk assessment.

Ten patients were identified through clinical samples (five in the old and five in the new building), six before (five within the past 6 months, one 18 months prior to hospitalization) and four during admission. However, 41 (80.4%) patients were not detected before or during hospitalization. The six patients that were already known to be a carrier of ESBL-E based on previous clinical cultures, had an electronic flag in their EHR and were cared for in isolation. Patients found to be ESBL-E positive during admission through clinical cultures, received an electronic flag during admission and were subsequently placed in isolation. Of the 41 unidentified carriers (6.9% of the 597 included patients), eleven out of 225 (4.9%) carriers were admitted to the old building and 30 out of 372 (8.1%) to the new building. Twenty-seven (65.8%) patients were positive at admission, of which 16 (61.5%) patients were also positive at discharge. Fourteen (34.2%) patients were only positive at discharge.

## Discussion

In this prospective before-and-after study, we could not show that transitioning from a hospital facility with multi-occupancy rooms to a new hospital building with 100% single-occupancy rooms significantly decreases ESBL-E acquisition. However, as a result from this relocation to 100% single rooms, we did observe a significant decrease in the number of intra-hospital patient transfers, which was associated with higher odds on ESBL-E acquisition, and a significant decrease in exposure to m^2^. WGS showed that most patients that carried an ESBL-E at admission and discharge carried indistinguishable strains. Finally, we showed a high proportion of unknown ESBL-E carriers, of which the majority was already ESBL-E positive at admission.

Only a small number of studies have determined the effect of single-occupancy rooms on HRMO acquisition, with conflicting results [10, 11, 19, 20]. The only study determining the effect of single-occupancy rooms on ESBL-E acquisition was performed by Levin et al. [11]. They determined that transitioning from an open plan ICU to single-occupancy rooms did not significantly decrease ESBL-E acquisition, which is similar to our results. Both Vietri et al. [19] and Ellison et al. [20], who looked at methicillin-resistant *Staphylococcus aureus* (MRSA) colonization and HAI with MRSA or vancomycin-resistant enterococci (VRE) respectively, found no difference after the transition to mainly single-occupancy rooms. However, our hospital transitioned to 100% single-occupancy rooms. The study of McDonald et al. [10] is the only study who also determined the effect of transitioning to a newly constructed hospital with 100% single-occupancy rooms. They determined the effect on MRSA and VRE colonization and infection, and on *Clostridioides difficile* infection (CDI) rates and observed that the transition did not impact CDI or MRSA infection rates, but did significantly decrease VRE colonization and infection rates and MRSA colonization rates [10]. Their results indicate that transitioning to 100% single-occupancy rooms can still positively impact the acquisition of other HRMO [10].

After relocating to the new hospital building, and thus after the transition to 100% single-occupancy rooms, the number of intra-hospital patient transfers decreased significantly. The biggest decrease was seen for hematological patients. Even though hematology wards already consisted of mainly single-occupancy rooms, patients in the old hospital were often first admitted to a multiple-occupancy room, and later transferred to a single-occupancy room. Additionally, we showed an association between intra-hospital transfers and ESBL-E acquisition, with higher odds for patients who were transferred at least twice. However, there could be other explanations for these increased odds, since the need for intra-hospital patient transfers could indicate the need for additional care. Consequently, these patients might have had contact with more healthcare workers, potentially had more intravenous or arterial catheters, and a higher antibiotic consumption, which are all potential risk factors for ESBL-E acquisition. Due to the small number of patients who acquired ESBL-E, we were unable to correct for these factors. An additional benefit of the reduction of transfers could be a reduction in workload, a decrease in cost, and a decrease in medical errors [8, 21–24].

As a result of the decrease in intra-hospital patient transfers, patients were exposed to less square meters of hospital environment in the new hospital building. Important is that not the intra-hospital patient transfers in itself, but the exposure to more, and different areas of, the hospital environment is a potential source for ESBL-E. However, since the exposure to the hospital environment is related to intra-hospital transfer, the number of intra-hospital transfers during hospitalization is an important risk factor for acquisition and should be included in future studies. In 19.8% of published outbreaks, the hospital environment was identified as the source [25]. Additionally, studies have shown increased odds on HRMO acquisition when the prior room occupant was infected/colonized [26]. While single-occupancy rooms in our hospital are cleaned after a patient is discharged, rooms are only disinfected when a known HRMO carrier was admitted to the room. Our study identified a high percentage of unknown ESBL-E carriers, highlighting the fact that HRMO carriers are missed. Consequently, some rooms are only cleaned when disinfection would have been appropriate, potentially leaving HRMO reservoirs behind. Therefore, a decrease in exposure to the environment, means less exposure to pathogenic organisms of other patients. Since the exposure to the environment is an important factor for HRMO acquisition, the impact of the transition to single-occupancy rooms on the m^2^ patients were exposed to is an important outcome of this study.

While the majority of patients positive both at admission and discharge had indistinguishable strains, for two patients the discharge strain was not identical to the admission strain. This can be explained by acquisition of a different strain during hospitalization, or by carriage of multiple strain types, of which only one was detected at admission. To identify possible different strain types, or species, with ESBL-genes, it is recommended to pick and analyze multiple colonies, even when they are morphologically identical. Interspecies plasmid transfer in the gut is possible through plasmid carriers, which could possibly lead to phenotypic resistance, among which the ESBL phenotype. However we did not perform plasmid analyses in the strains from these two patients.

We determined a prevalence of ESBL-E at admission of 4.4% in the old building, and 6.5% in the new building, which is in agreement with previous reports on the prevalence of ESBL-E in the Netherlands, with ranges between 4.5% and 8.6% in 2018 [27–29]. Of the 51 identified ESBL-E carriers, 34 were positive upon admission. The majority of these patients had no recent hospitalizations, suggesting that the majority of ESBL-E was community acquired. Twelve carriers were only positive at admission, indicating loss of the ESBL-E during hospitalization. A possible explanation is that they received antibiotic therapy during hospitalization, however, it is also possible that these were false-negative results. The high number of unidentified ESBL-E carriers can partly be explained by the fact that the risk-assessment questions asked at admission were unable to identify 49 out of the 51 (96.1%) ESBL-E carriers. Six of the 49 patients had already an electronic label in the EHR as being an ESBL-E carrier due to previous ESBL-E positive cultures and were thus known carriers to the hospital regardless of the risk-assessment outcome. Van Hout et al. [18] compared the observed prevalence of ESBL-E carriers newly identified via the risk assessment to the perceived ESBL-E carriage rate based on epidemiological studies in the Netherlands. They determined that the risk-assessment identified less than 1% of all ESBL-E carriers [18]. A case control study in MRSA carriers without known risk factors found previously unknown risk factors, explaining 83% of the MRSA of unknown origin [30]. Bastiaens et al. [31] identified that active surveillance in patients hospitalized for ≥ 14 days can be used to identify asymptomatic HRMO colonization. Even though this added screening can help identify previously unknown carriers, after 14 days transmission to other patients or the environment could have already occurred within the hospital. Therefore, it should also be considered to determine additional risk factors for ESBL-E carriage, for example questions about travel history [32–35] or antibiotic usage in the last 90 days, specifically targeting use of fluoroquinolones and beta-lactams [29, 33, 36]. An improved risk-assessment could help decrease the number of unidentified carriers at admission and hence prevent transmission to other patients within the hospital.

## Strengths and limitations

The main strength of our study was that the relocation of the hospital provided us the opportunity to determine the difference in risk on acquisition of ESBL-E between multiple-occupancy rooms and single-occupancy rooms for patients from different departments and specializations. Additionally, performing WGS analyses provided us additional insights in ESBL-E colonization compared to only microbiological culture methods.

However, our study also has some limitations. The most important limitation was the low prevalence of ESBL-E, and the low incidence of ESBL-E acquisition. As a result, we did not have enough statistical power to perform multivariate analyses and were thus unable to correct for possible confounding factors, such as differences in hand hygiene compliance and cleaning protocols. Since we did not perform a sample-size calculation before the start of the study, it is possible that our study is underpowered. Additionally, we used perianal samples instead of rectal samples. While perianal swabs are less invasive then rectal swabs and might increase participation, it is known that the sensitivity of perianal swabs is lower compared to rectal swabs [37]. By using selective broths and culture methods, we aimed to minimize the risk of false-negative results, but it is likely that ESBL-E carriers and ESBL-E acquisitions were missed. Additionally, repeated sampling throughout the hospitalization period would also have decreased the chance for false-negative samples. A final sampling limitation is that patients missed at discharge were asked to sample at home, which meant a delay in sampling. Therefore, not all discharge samples might be representative of the situation at discharge. Furthermore, we have introduced selection bias as a consequence of our inclusion criteria, and by the fact that the proportion of patients admitted to the different specializations was different in the old building compared to the new hospital building. An explanation for this is the fact that after the relocation of hematology patients from the Cancer Institute to the new hospital building, it was easier to approach and hence include these patients. Finally, we did not include all patients admitted to the participating departments. Therefore, we were unable to determine the exact dynamics of ESBL-E within and between departments.

## Conclusion

Due to the design of the study, a significant decrease in ESBL-E acquisition after relocating to the new hospital could not be shown, but the transition to a hospital with 100% single-occupancy rooms was associated with a significant decrease in intra-hospital patient transfers and, hence, a significant decrease in exposure to square meters. By determining that transferred patients had higher odds on ESBL-E acquisition, we showed that the transition to 100% single-occupancy rooms can indirectly impact ESBL-E acquisition. Additionally, the large proportion of ESBL-E carriers that remains unidentified by clinical samples highlights the need for an improved risk-assessment screening at admission. Future research is needed to determine the impact of 100% single occupancy rooms on factors that could impact ESBL-E and HRMO acquisition, such as exposure to square meters as a measure for exposure to the hospital environment, and to develop an effective risk-assessment screening.

## Supplementary Information

**Additional file1** Calculating the square meters

**Additional file2 ** Detected AMR genes and heatmaps for ESBL-producing E. coli and K. pneumoniae

**Additional file3 ** HRMO screening risk assessment questions upon admission to the hospital

## Abbreviations

95%CI: 95% Confidence interval
AMR: Antimicrobial resistance
CDI: *Clostridium difficile* Infection
cgMLST: Core genome multi locus sequence type
EHR: Electronic health records
Erasmus MC: Erasmus MC university medical center
ESBL: Extended-spectrum beta-lactamase
ESBL-E: Extended-spectrum beta-lactamase-producing Enterobacterales
EUCAST: European committee on antimicrobial susceptibility testing
HAI: Healthcare associated infections
HRMO: Highly resistant microorganisms
ICU: Intensive care unit
m^2^: Square meters
MALDI-TOF: Matrix-assisted laser desorption/ionization time-of-flight mass spectrometry
MRSA: Methicillin-resistant *Staphylococcus aureus*
OR: Odds ratio
SPSS: Statistical package for the social sciences solutions
ST: Sequence type
VRE: Vancomycin-resistant enterococci
WGS: Whole genome sequencing

## Declarations

### Availability of data and materials

The datasets generated and/or analyzed during the current study are not publicly available due to privacy of the participating patients but are available from the corresponding author on reasonable request.
