# Correction: The effect of 100% single-occupancy rooms on acquisition of extended-spectrum beta-lactamase-producing Enterobacterales and intra-hospital patient transfers: a prospective before-and-after study

**DOI:** 10.1186/s13756-022-01205-9

**Published:** 2022-12-19

**Authors:** Adriënne S. van der Schoor, Juliëtte A. Severin, Anna S. van der Weg, Nikolaos Strepis, Corné H. W. Klaassen, Johannes P. C. van den Akker, Marco J. Bruno, Johanna M. Hendriks, Margreet C. Vos, Anne F. Voor in ’t holt

**Affiliations:** 1 Department of Medical Microbiology and Infectious Diseases, Erasmus MC University Medical Center, Rotterdam, The Netherlands; 2 Department of Intensive Care Adults, Erasmus MC University Medical Center, Rotterdam, The Netherlands; 3 Department of Gastroenterology and Hepatology, Erasmus MC University Medical Center, Rotterdam, The Netherlands; 4 Department of Surgery, Erasmus MC University Medical Center, Rotterdam, The Netherlands

**Correction to: Antimicrob Resist Infect Control (2022) Jun 2;11(1):76.** **https://doi.org/10.1186/s13756-022-01118-7**.

The authors wish to make the following corrections to the article. First, the original article [1] contained an error in Table 2. Two isolates reported as *Klebsiella pneumoniae* proved to be *Klebsiella aerogenes*. Additionally, isolates identified as *Proteus vulgaris* involved different *Proteus* species. Second, discrepancies were identified in the results of the whole genome sequencing analyses presented in Additional file 2. These were corrected, and the description of the methods were adjusted accordingly. The correct table, methods, and Additional file 2 are located ahead in this Correction article and should be considered instead.

**Table 2:** Number of patients who were positive for ESBL-producing Enterobacterales at admission, at discharge, and the number of patients who acquired an ESBL-producing Enterobacterales

	Old hospital building (n = 225)			New hospital building (n = 372)		
	Admission (%)	Discharge (%)	Acquisition (%)^4^	Admission (%)	Discharge (%)	Acquisition (%)
No ESBL-E	215 (95.6)	214 (95.1)	NA	348 (93.5)	344 (92.5)	NA
ESBL-E^1,2^	10 (4.4)	11 (4.9)	7 (3.1)	24 (6.4)	28 (7.5)	13 (3.2)
*Escherichia coli*^*3*^	6 (2.7)	8 (3.5)	5 (2.2)	19 (5.1)	22 (5.9)	8 (2.2)
*Klebsiella pneumoniae*	1 (0.4)	3 (1.3)	2 (0.9)	1 (0.3)	4 (1.1)	3 (0.8)
*Citrobacter freundii*	2 (0.9)	0 (–)	NA	0 (–)	1 (0.3)	1 (0.3)
*Proteus* spp.^5^	1 (0.4)	0 (–)	NA	2 (0.5)	0 (–)	NA
*Enterobacter cloacae* complex	0 (–)	0 (–)	NA	1 (0.3)	0 (–)	NA
*Morganella morganii*	0 (–)	1 (0.4)	1 (0.4)	0 (–)	0 (–)	NA
*Klebsiella aerogenes*	0 (–)	0 (–)	NA	1 (0.3)	2 (0.5)	1 (0.3)

*ESBL* extended-spectrum beta-lactamase, *ESBL-E* extended-spectrum beta-lactamase producing Enterobacterales, *NA* not applicable

^1^Five patients in the old building, and seven patients in the new building were ESBL-E positive at admission and ESBL-E negative at discharge

^2^Non-significant difference between the old hospital setting and the new hospital setting for admission (*P* = 0.305), for discharge (*P* = 0.206), and for acquisition (*P* = 0.801)

^3^Non-significant difference between the old hospital setting and the new hospital setting for admission (*P* = 0.149), for discharge (*P* = 0.156), and for acquisition (*P* = 0.901)

^4^One patient was positive at admission but acquired a different ESBL-E during hospitalization and one patient acquired two ESBL-E in the old building. Consequently, there are seven patients who acquired an ESBL-E during hospitalization in the old building, but eight different ESBL-E

^5^One *Proteus faecis,* one *Proteus terrae*, and one unknown *Proteus* spp

## Methods

### Whole genome sequencing

WGS was performed for all identified ESBL-E isolates. Total genomic DNA was extracted using the MagNA Pure 96 platform (Roche Applied Science, Mannheim, Germany). Genomic DNA was fragmented by shearing to a size of ~ 350 bp. Libraries were prepared using the NEBNext® DNA Library Prep kit (New England Biolabs, Ipswich, MA, USA) and subjected to 150 bp paired-end sequencing creating > 100 × coverage using Illumina technology (Novogene, HongKong, China). De novo genomic assemblies were generated using CLC Genomics Workbench v21 (Qiagen, Hilden Germany) using default parameters. Antimicrobial resistance (AMR) genes were detected and identified using the web-based Comprehensive Antibiotic Resistance Database (CARD) interface (https://card.mcmaster.ca/) restricted to perfect and strict hits [16]. Conventional multi locus sequence types (MLST) and core-genome MLST cluster types were determined using each species’ corresponding scheme (https://cgmlst.org/ncs) in SeqSphere + v5 software (Ridom, Munster, Germany). The identity of all strains was verified by analyzing the genomic assemblies using the online TYGS platform (https://tygs.dsmz.de/) [17].

## Supplementary Information

**Additional file 2**. Detected AMR genes and heatmaps for ESBL-producing *Escherichia coli* and *K pneumoniae*
