# Supplementary material for: The effect of 100% single-occupancy rooms on acquisition of extended-spectrum beta-lactamase-producing Enterobacterales and intra-hospital patient transfers: a prospective before-and-after study
Source: Antimicrob Resist Infect Control. 2022 Jun 2;11:76. doi: 10.1186/s13756-022-01118-7 (PMC9164559; doi:10.1186/s13756-022-01118-7)
Supplement: Supplementary file 1 — Additional file1 Calculating the square meters [file 13756_2022_1118_MOESM1_ESM.docx]

**Additional file 1:** Calculating the square meters

To determine the surface area patients were exposed to, the square meters of the rooms were calculated. First, we standardized the square meters of patient rooms, because small differences in square meters did occur between different wards. The standardized surfaces we used, were:

Old hospital building:

- Four-person room, excluding bathroom shared with ward mates: 46.83 m^2^
- Two-person room, excluding bathroom shared with ward mates: 17.75 m^2^
- Bathroom (i.e. toilet and shower) on the ward: 6.50 m^2^
- Single-occupancy room hematology, including private bathroom: 26.21 m^2^
- Two-person room hematology, including bathroom shared with roommates: 22.12m^2^
- Three-person room hematology, including bathroom shared with roommates: 43.62m^2^

New hospital building:

- Single-occupancy room including private bathroom: 26.21 m^2^

To calculate the total square meters a patient was exposed to during hospitalization, we included all rooms a patient was admitted to according to his/her electronic health records. In the old building bathrooms were shared with ward mates, with multiple available toilets and showers. When a patient was moved to a different room on the ward, but close to the previous room, no additional square meters for the bathroom were added. When a patient was relocated to the other side of the ward, we assumed that the patient would use a different bathroom then before and thus added additional square meters.

**Results per specializations**

In the new building, patients were exposed to less m^2^ during hospitalization than in the old building. Overall, the median m^2^ patients were exposed to in the old building was 43.3m^2^ (21.9-177.9), compared to 22.9m^2^ (22.9-114.6) in the new building (*P*<0.001). The median m^2^ was significantly lower for all medical specializations. For medical patients, the median m^2^ decreased from 52.4 m^2^ (22.9-77.9) in the old building to 26.6 m^2^ (22.9-68.7) in the new building (*P*<0.001), for surgical patients from 52.4 m^2^ (22.9-77.9) in the old building to 26.6 m^2^ (22.9-68.7) in the new building (*P*<0.001), and for hematological patients from 48.1 m^2^ (21.9-118.2) to 24.9 m^2^ (22.9-68.7) (*P*<0.001).
