# Supplementary material for: The effect of 100% single-occupancy rooms on acquisition of extended-spectrum beta-lactamase-producing Enterobacterales and intra-hospital patient transfers: a prospective before-and-after study
Source: Antimicrob Resist Infect Control. 2022 Jun 2;11:76. doi: 10.1186/s13756-022-01118-7 (PMC9164559; doi:10.1186/s13756-022-01118-7)
Supplement: Supplementary file 2 — Additional file2 Detected AMR genes and heatmaps for ESBL-producing E. coli and K. pneumoniae [file 13756_2022_1118_MOESM2_ESM.docx]

**Additional file 2**: Detected AMR genes and heatmaps for ESBL-producing *E. coli* and *K. pneumoniae*

All *E. coli* isolated carried *bla*_ampC_, and *bla*_CTX-M_ (different sub types), and some isolates carried *bla*_CMY(132-like)_, *bla*_TEM-1_, *bla*_OXA-1_ and *bla*_OXA-10_. No differences in AMR genes were identified between admission and discharge *E. coli* strains, with the exception of three patients. The discharge strain of patient 172 had the same cgMLST sequence type, but was not identical, with *bla*_OXA-1_ and *aadA5* present only at discharge. The discharge strain of patient 682 obtained *fosA5* and ANT(3’’)-IIa, while three AMR related efflux pump genes (*acrA*, *yojl*, and *tolC*) were not detected. The discharge strain of patient 773 was the same isolate, however, it carried an additional *bla*_oxa-1_ and AAC(6’)-Ib-cr genes. The discharge strain of patient 14 was not identical to the admission strain, but no differences in AMR genes were observed. The majority of *K. pneumoniae* strains contained *bla*_CTX-M-15_, *bla*_TEM-1_, *bla*_SHV_ and some strains contained *bla*_OXA-1_. No differences in AMR genes were identified between the admission and discharge *K. pneumoniae* strains.

Of the 16 patients included twice during the study period, two patients were positive for ESBL-E. Patient 128 was positive for an ESBL-producing *E. coli* at admission and discharge for both admissions. All strains were identical according to cgMLST, and no differences were observed in AMR genes. Patient 5 was positive for an ESBL-producing *Citrobacter freundii* at admission, but for an ESBL-producing *K. pneumoniae* at discharge. For the second hospitalization, the patient was positive for an ESBL-producing *K. pneumoniae* at admission and discharge.

**Additional figure 1.** Heatmap for *E. coli* isolates of patients positive at admission and discharge. Strains from patients are observed to form clusters based on their similarities on presence of the antimicrobial resistance genes. Blue represents a perfect hit, teal indicates a strict hit while blank shows absence of the AMR gene based on CARD analysis.

**Additional figure 2.** Heatmap for *K. pneumoniae* isolates of patients positive at admission and discharge. Strains from patients are observed to form clusters based on their similarities on presence of the antimicrobial resistance genes. Blue represents a perfect hit, teal indicates a strict hit while blank shows absence of the AMR gene based on CARD analysis.

**Additional figure 3.** Core genome MLST analysis based on 2358 loci of *K. pneumoniae* isolates from patients positive at admission and discharge, and of *K. pneumoniae* isolates acquired during hospitalization. Node numbers represent isolate numbers and line numbers show the number of different alleles between the isolates. A cut-off value of >15 alleles difference (embedded in the SeqSphere software) was applied to consider strains to be different. Colors match the sequence types (ST). A: Admission, D: Discharge and Ac: Acquired.
