# Supplementary material for: The effect of 100% single-occupancy rooms on acquisition of extended-spectrum beta-lactamase-producing Enterobacterales and intra-hospital patient transfers: a prospective before-and-after study
Source: Antimicrob Resist Infect Control. 2022 Jun 2;11:76. doi: 10.1186/s13756-022-01118-7 (PMC9164559; doi:10.1186/s13756-022-01118-7)
Supplement: Supplementary file 3 — Additional file3 HRMO screening risk assessment questions upon admission to the hospital [file 13756_2022_1118_MOESM3_ESM.docx]

**Additional file 3: HRMO screening risk assessment questions upon admission to the hospital**

1. Is the patient/family/counselor available to answer questions?
2. Has the patient recently been treated in or admitted to a foreign healthcare institution?
3. Does the patient live or work where pigs, veal calves or broilers are kept commercially?
4. Is the patient a known carrier of an HRMO?
5. Is the patient a partner, housemate or caretaker of someone who is MRSA positive?
6. Did the patient stay in a healthcare facility known with an HRMO outbreak in the past 2 months, and if yes was the patient approached for screening?
7. Has the patient lived in an institution for asylum seekers in the past 2 months?
8. Is the patient a professional seafarer?
