# Supplementary material for: Correction: The effect of 100% single-occupancy rooms on acquisition of extended-spectrum beta-lactamase-producing Enterobacterales and intra-hospital patient transfers: a prospective before-and-after study
Source: Antimicrob Resist Infect Control. 2022 Dec 19;11:162. doi: 10.1186/s13756-022-01205-9 (PMC9764685; doi:10.1186/s13756-022-01205-9)
Supplement: Supplementary file 1 — Additional file 2. Detected AMR genes and heatmaps for ESBL-producing Escherichia coli and K pneumoniae [file 13756_2022_1205_MOESM1_ESM.docx]

**Additional file 2**: Detected AMR genes and heatmaps for ESBL-producing *Escherichia coli* and *K pneumoniae*

Of the 16 patients included twice during the study period, two patients were positive for ESBL-E. Patient 128 was positive for an ESBL-producing *E. coli* at admission and discharge for both admissions. These *E. coli* strains were identical according to cgMLST, and no differences were observed in AMR genes. Patient 5 was positive for an ESBL-producing *Citrobacter freundii* at admission, but for an ESBL-producing *K. pneumoniae* at discharge. For the second hospitalization, the patient was positive for an ESBL-producing *K. pneumoniae* at admission and discharge.

Details for isolates of *E. coli* (A) and *K. pneumoniae* (B), including patient number, sampling moment, conventional MLST results, cgMLST cluster types determined by SeqSphere+ software (Ridom, Munster, Germany) and presence of antibiotic resistance genes (search restricted to perfect and strict matches) as determined using the CARD web-interface (https://card.mcmaster.ca/). Results shown are focused on different types of beta-lactamases and aminoglycoside modifying enzymes. A grey background indicates patients with strains having different CARD results between admission and discharge despite having an isogenic chromosomal background. A yellow background indicates patients with strains of a different genetic background between admission and discharge. Pink indicates patients only testing positive at discharge. A black background indicates presence of the gene. Numbers indicate percentage identity to the CARD reference sequence.
